# Challenges Associated With the Design and Deployment of Food Intake Urine Biomarker Technology for Assessment of Habitual Diet in Free-Living Individuals and Populations—A Perspective

**DOI:** 10.3389/fnut.2020.602515

**Published:** 2020-11-25

**Authors:** Manfred Beckmann, Thomas Wilson, Amanda J. Lloyd, Duarte Torres, Ana Goios, Naomi D. Willis, Laura Lyons, Helen Phillips, John C. Mathers, John Draper

**Affiliations:** ^1^Institute of Biological, Environmental and Rural Sciences, Aberystwyth University, Aberystwyth, United Kingdom; ^2^Faculty of Nutrition and Food Sciences, University of Porto, Porto, Portugal; ^3^Epidemiology Research Unit (EPIUnit), Institute of Public Health, University of Porto, Porto, Portugal; ^4^Human Nutrition Research Centre, Population Health Sciences Institute, William Leech Building, Newcastle University, Newcastle-upon-Tyne, United Kingdom

**Keywords:** dietary intake, metabolomics, biomarker of food intake (BFI), urinary biomarkers, habitual diet

## Abstract

Improvement of diet at the population level is a cornerstone of national and international strategies for reducing chronic disease burden. A critical challenge in generating robust data on habitual dietary intake is accurate exposure assessment. Self-reporting instruments (e.g., food frequency questionnaires, dietary recall) are subject to reporting bias and serving size perceptions, while weighed dietary assessments are unfeasible in large-scale studies. However, secondary metabolites derived from individual foods/food groups and present in urine provide an opportunity to develop potential biomarkers of food intake (BFIs). Habitual dietary intake assessment in population surveys using biomarkers presents several challenges, including the need to develop affordable biofluid collection methods, acceptable to participants that allow collection of informative samples. Monitoring diet comprehensively using biomarkers requires analytical methods to quantify the structurally diverse mixture of target biomarkers, at a range of concentrations within urine. The present article provides a perspective on the challenges associated with the development of urine biomarker technology for monitoring diet exposure in free-living individuals with a view to its future deployment in “real world” situations. An observational study (*n* = 95), as part of a national survey on eating habits, provided an opportunity to explore biomarker measurement in a free-living population. In a second food intervention study (*n* = 15), individuals consumed a wide range of foods as a series of menus designed specifically to achieve exposure reflecting a diversity of foods commonly consumed in the UK, emulating normal eating patterns. First Morning Void urines were shown to be suitable samples for biomarker measurement. Triple quadrupole mass spectrometry, coupled with liquid chromatography, was used to assess simultaneously the behavior of a panel of 54 potential BFIs. This panel of chemically diverse biomarkers, reporting intake of a wide range of commonly-consumed foods, can be extended successfully as new biomarker leads are discovered. Towards validation, we demonstrate excellent discrimination of eating patterns and quantitative relationships between biomarker concentrations in urine and the intake of several foods. In conclusion, we believe that the integration of information from BFI technology and dietary self-reporting tools will expedite research on the complex interactions between dietary choices and health.

## Introduction

There is a rich history of nutrition research spanning many decades, much of which has had at its core a need for accurate information on dietary intake for investigation of the links between exposure to individual food/food groups and specific health outcomes. Food intervention projects commonly rely on participants collecting pre-prepared foods from research centres for consumption at home and then confirming compliance at a later date (1, 2). On the other hand, large-scale nutritional epidemiological projects and nutrition surveys involving free-living individuals consuming their habitual diet rely almost totally on self-reporting of dietary exposure. Long-established tools to collect self-reported quantitative dietary information include Food Frequency Questionnaires (FFQs), diet diaries, and dietary recall methodology (3). However, because of the complexity of eating patterns and the conceptual and practical difficulties in recording or recalling the types and amounts of foods and beverages consumed, errors in self-reporting of dietary intakes by cognitively-able individuals is commonplace and substantial (4, 5) and can be exacerbated in those who are overweight or obese (6, 7).

Secondary metabolites derived from individual foods or food groups present in human biofluids can provide potential biomarkers of food intake, for reviews see (8–18). The inclusion of biomarker technology in dietary assessment could help to overcome some of the limitations of traditional dietary methodologies by providing additional objective estimates of food exposure (19). Unlike blood, urine is easy to collect and it provides an integrated estimate of exposure over several hours. For a panel of dietary biomarkers to have any significant utility, it is essential that its coverage is as comprehensive as possible. Using data-driven approaches, we have shown that the potential utility of a biomarker is dependent on the type, portion size, and frequency of consumption of individual foods (20). Data concerning nationally-representative estimates of intakes of foods by the UK population are collected by the UK National Diet and Nutrition Survey (NDNS) (21) and this database can be explored to identify foods and food groups for which dietary exposure biomarker discovery might be feasible and relevant (1, 2, 22).

Over the past decade, our collaborative research projects and those of other teams (see Supporting Data 1 for a comprehensive list) have contributed to the discovery of putative dietary intake urinary biomarkers of specific foods including poultry and red meat (23–28) citrus fruits (29, 30), crucifers (31, 32), oily fish (26, 27, 32), red berries/strawberries (2, 32–34), wholegrain/rye (35–37), sugary drinks (38, 39), artificial sweeteners (2, 40), peas/beans/legumes (2, 41, 42), grapes (41, 43–45), apples (41, 46, 47), and potatoes (48). In addition, consensus guidelines for the critical assessment of candidate BFIs has been established (49). These BFI candidate guidelines have focused generally on qualifying the utility of individual BFIs for monitoring exposure to specific foods/food groups. However, because effects on health are a consequence of the whole diet, it is equally important to develop approaches to assess overall dietary exposure in nutrition surveys, epidemiological studies, and clinical trials (45).

The ideal biomarker is highly specific for one food item or food group, is not detected in the biological sample of interest when the specific food item is not ingested, and shows a distinct dose- and time-dependent response following consumption (50). Although metabolites distinctive of dietary exposure to particular foods have been described, it is not uncommon to discover subsequently that putative biomarkers are not necessarily specific for individual foods and therefore much rigour needs to be applied during validation of their utility to monitor habitual dietary intake (51). For application in the real world, the use of multi-metabolite biomarker panels may provide more reliable estimation of dietary exposure than a single-biomarker approach [reviewed by (52)]; such panels need to have comprehensive coverage and to be extendable (53). For this reason, in the future it will be important to evaluate biomarker performance in the context of complex exposures to multiple foods, with different food formulations, cooking, and processing methods and within complex meals, in eating patterns the target study population is likely to experience (2).

Optimal sampling requirements for urine biomarker analysis will be dependent largely on the study design and objectives (Table 1). For example, a food intervention study with free-living participants lasting several weeks investigating links between a health outcome and a specific food/food group will require appropriate samples on multiple days taken at random to assess compliance with dietary intake targets (1, 2). In contrast, assessing the general eating habits of a large population in an epidemiological survey may only require sampling of a large number of people on a single random day or multiple days (60). Any urine sampling procedure would need to be (i) acceptable for volunteers to provide samples repeatedly, (ii) require minimal researcher time and cost, and (iii) deliver samples with high quality information content. The theoretical optimal types of urine(s) to be sampled [e.g., spot, cumulative (i.e., “phase” of day) or 24 h] will also depend on study objectives (62) and, in many instances, the sampling strategy will be limited by cost constraints or the practicalities of collection. Twenty-four hour urine samples and single spot urine samples taken at random times during the day are commonly collected to monitor discrete aspects of human physiology, metabolism, or “exposome” in clinical trials and surveys (63–65). Unfortunately, such samples provide information only in relation to very recent eating behaviour and may be of limited utility in nutrition studies where the focus is on the whole diet or on the intakes of foods that are not eaten frequently. Additionally, eating behaviour and hydration levels can be very different between individuals in free-living populations and the fact that excretion half-lives of specific metabolites can vary enormously (49), means that research protocols must be in place to manage adequately these sources of variability in any biomarker discovery and validation strategy (62, 66).

**Table 1:** Study objectives and biomarker of food intake (BFI) requirements.

**Example study objectives**		**Typical sampling requirements**		**Biomarker requirements**		**Data requirements**		**Study example and reference**
		**Single sample only**	**Multiple samples**	**Biomarker(s) of only one food/food group**	**Comprehensive biomarker panel**	**Quantitative or semi-quantitative measurement**	**Exposure range assignment**	
A	Confirmation of participant compliance in a food intervention study or validation of a proposed biomarker focusing on a single food/food group, short term or long term		Y	Y		Y		A validation trial: (54)A compliance trial: (55, 56)
B	Biomarker discovery and/or validation in a free-living population following a meal plan emulating normal eating patterns		Y		Y	Y		MAIN study: (1, 2, 22)
C	Investigation of individual “metabotype” in relation to interaction with specific dietary chemicals	?	?	Y		Y		Food4me study: (57)
D	Assessment of habitual (e.g., weekly, monthly, and annual) eating behavior of individuals		Y		Y	?	?	PREDIMED trial: (58, 59)
E	Observational epidemiological survey of eating habits in a large population	Y			Y	Y	?	IAN-AF: (60); EPIC: (27, 61)
F	Cohort stratification by dietary exposure levels to specific foods/food groups in a small clinical trial		Y		Y		Y	MAIN study: (45)

Where the aim is to estimate absolute intake of specific foods, or the frequency of exposure, it will be desirable to generate quantitative or semi-quantitative data on BFI concentrations in urine. However, for other studies, it may be sufficient to be able to assign each individual into an exposure range (e.g., high-medium-low), typical of a specific reference population. Urine collection(s), sample processing, and the analytical methodology can be optimised for a target metabolite when using a single biomarker to monitor exposure to a single food/food group. In contrast, the desire to monitor habitual diet comprehensively using a panel of biomarkers requires the analytical approach to manage the complex physio-chemical attributes of the diverse range of putative biomarkers currently described, as well as coping with metabolites exhibiting differential stability during collection, transport, and storage. Other practical issues such as the commercial availability, costs, solubility, and stability of pure chemicals in mixtures as quantitation standards will also impact on the design of analytical solution likely to offer scope for simultaneous measurement of a large number of metabolite targets.

The assessment of eating behaviour in free-living individuals is important in a wide range of types of nutrition research (Table 1), ranging from clinical trials investigating the mode of action of potentially beneficial “bioactive” compounds in individuals, to general surveys of national eating habits in large populations. Although considerable effort is being expended on BFI discovery and validation, there is an equally urgent need to consider the future challenges for effective deployment of dietary intake biomarker technology to assess habitual diet within populations. To summarise, these major challenges include:

- Strategies for validation of food intake biomarkers suitable for assessment of habitual dietary exposure;
- Standardised urine sampling approaches, including collection, temporary storage, transport, and long term biobanking;
- Development of biomarker analytical methodology, using a multi-panel of biomarkers, that is able to integrate new markers as they become validated;
- Algorithms to convert raw biomarker data into meaningful estimates of food intake and/or overall diet quality.

**Figure 1:**
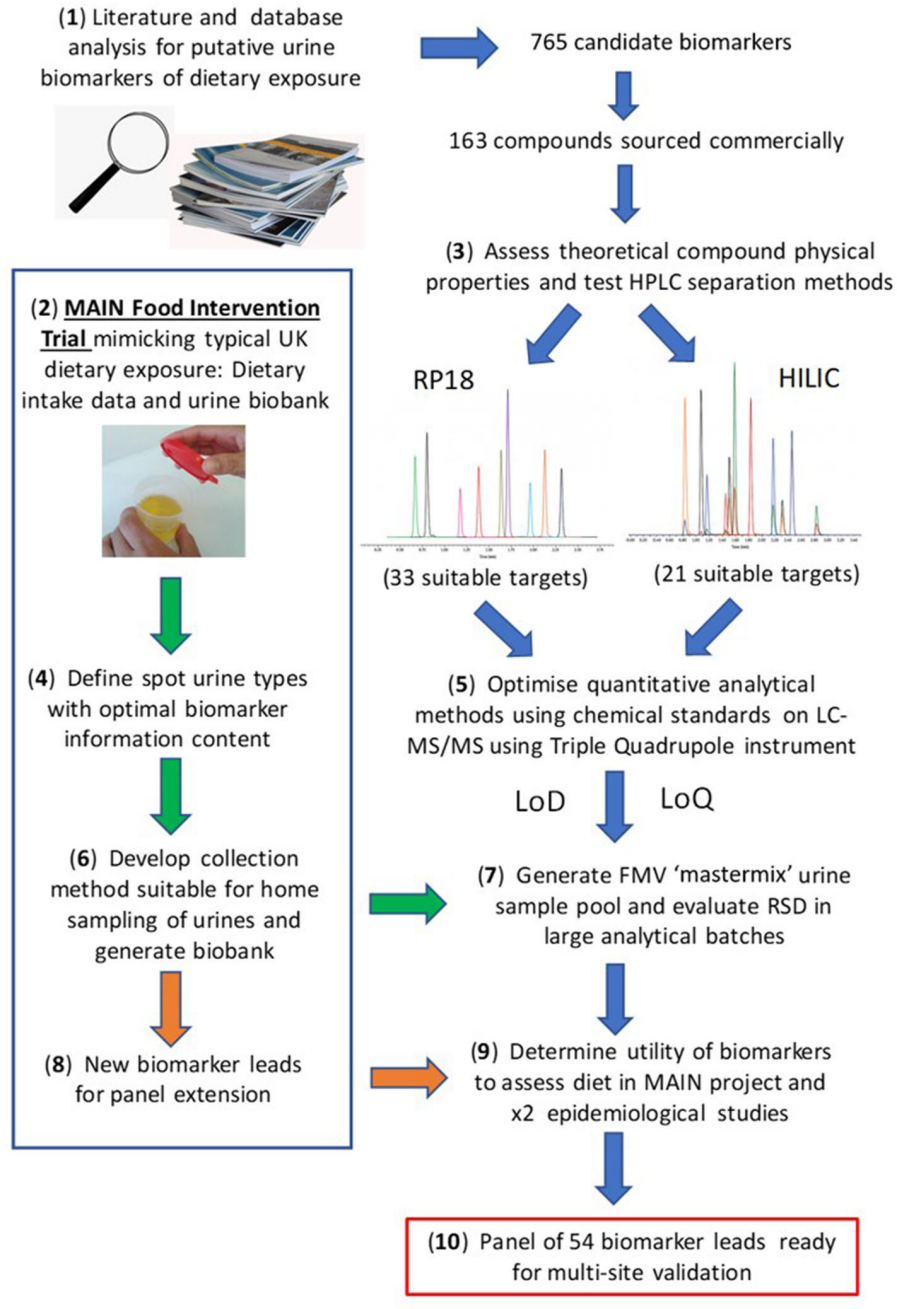
Workflow for biomarker panel development. Where: MAIN, Metabolomics at Aberystwyth, Imperial and Newcastle; RP, reverse phase; HILIC, Hydrophilic Interaction Liquid Chromatography; LoD, limit of detection; LoQ, limit of quantification.

The present article aims to provide a perspective on *some* of these challenges associated with the development of urine biomarker technology to monitor recent or habitual dietary intake in free-living individuals with a view to its future deployment in “real world” situations. For example the UK government's “Better Health” campaign, a 12 week fitness and healthy eating plan announced in July 2020 to help Briton's lose weight and reduce their risk of serious complications should they contract COVID-19 (https://www.nhs.uk/better-health/). One of the key aims of the plan is to encourage people to make healthier food choices. We believe that the BFI technology we have been developing over the past 10 years, alongside the low-effort, minimally intrusive urine sampling strategies (62, 67) will soon be validated to the point that they can be used to reproducibly and objectively monitor the effectiveness of such plans at a population level. A workflow summarising the overall experimental strategy is illustrated in Figure 1.

## Materials and Methods

### Ethics Approval and Consent to Participate

For ***Study 1***, ethical approval was obtained from the National Commission for Data Protection, the Ethical Committee of the Institute of Public Health of the University of Porto and from the Ethical Commissions of each one of the Regional Administrations of Health. All participants gave written informed consent, and the study was carried out in accordance with the Declaration of Helsinki. The MAIN (Metabolomics at Aberystwyth, Imperial, and Newcastle) food intervention trial at Newcastle (***Study 2***) was approved by the East Midlands—Nottingham 1 National Research Ethics Committee (14/EM/0040). Caldicott approval for storage of data and data protection was granted by Newcastle-upon-Tyne Hospitals NHS Foundation Trust [6896(3109)]. The MAIN food intervention trial in Newcastle was adopted into the UK Clinical Research Network (CRN) Portfolio (16037) and is registered with International Standard Randomised Controlled Trials Number (ISRCTN), 88921234.

The participants provided written informed consent to participate in each study, taken by an appropriately trained researcher. All procedures performed in studies involving human participants were in accordance with the ethical standards of the institutional and/or national research committee and with the 1964 Helsinki declaration and its later amendments or comparable ethical standards.

### Epidemiological Study and Urine Sampling

***Study 1***involved community-living individuals consuming a freely-chosen diet. The participants (*n* = 95) were volunteers who participated in the Portuguese National Food, Nutrition, and Physical Activity Survey (IAN-AF), whose aims and methods have been described previously (60). A 24 h dietary record was collected by trained nutritionists using the “eAT24” Software (68) which facilitates the assessment of dietary data using an automated multiple-pass method (5 steps) (69). Participants were asked to collect urine samples on the day before the second 24 h dietary record. Urine samples were collected in two separate containers. The first one (a 2,700 mL container identified as container A) was used to collect all urine passed during the day before the interview, except the first void of that morning. A second one (a 500 mL container identified as container B) was used to collect just the First Morning Void (FMV) on the day of the second interview. No preservatives were added to the urine containers, and the participants were asked to keep the samples refrigerated (4°C) throughout the collection period. Participants were asked to fill in a questionnaire with the time of the beginning and the end of collections, details of any medication, and whether or not they had any problems or missed urine collections. At the laboratory, urine samples were weighed and mixed. The weights of urine from containers A and B were quantified separately and a proportionally pooled 24 h urine sample (identified as “24 h urine”) was prepared by using samples A and B. From each participant, both urine samples were aliquoted: 1 × 45 mL (in 50 mL Falcon pre-labelled tube) + 10 × 1.5 mL (in 2 mL pre-labelled microtubes). These aliquots were refrigerated immediately before being moved to −80°C storage, within 24 h, for further analysis.

### Food Intervention Study Design and Urine Sampling

The MAIN project at Newcastle included two controlled food intervention studies in free-living people who consumed the test foods as part of two 3day menu plans, designed to generate six distinctive “Menu Days” (1, 22). Participants were provided with all the foods and ingredients to prepare and consume meals at home, following the prescribed menus. Within this manuscript (***Study 2)***we have used data from 15 of the individuals from the second 3-day menu plan (8 male, 7 female; non-smokers; age: 21–74). We implemented urine sampling methods based on our previous studies (23, 70) and asked participants to collect a series of urine samples including the FMV the day after each menu plan. Participants collected urine samples in a plastic jug and transferred aliquots into labelled sterile 25 mL Universal tubes. Six of these 15 participants (2 female, non-smokers, age range 22–59) also collected FMV urine samples at home using the vacuum transfer system (67). All samples were placed in an opaque cool bag and stored at home in a fridge at 4°C for up to 4 days and then brought to the research facility in Newcastle at the end of the study week. Universal tubes were stored immediately at −80°C and the vacuum tubes remained at 4°C for a further 2 weeks before storage at −80°C. Samples were then transported to the analytical facility in Aberystwyth on dry ice for metabolite analysis.

### Urine Sample Preparation

Urine samples were prepared and adjusted as reported previously (1, 22). In brief, all urine samples were normalised by refractive index (RI) prior to analysis to account for differences in fluid intake by participants and to ensure that all Mass Spectrometry (MS) measurements were made within a similar dynamic range within the linear range of the instrument. Samples were defrosted overnight at 4°C, centrifuged (1,600 × g for 5 mins at 4°C), placed on ice and aliquots of thawed urine (1,000 μL) were transferred into labelled 2 mL Eppendorf tubes. The remaining sample were returned to a −20°C freezer. An OPTI Hand Held Refractometer (Bellingham Stanley™ Brix 54 Model) was used to record the specific gravity (SG). Using these data, aliquots of the required amounts of urine from centrifuged 2 ml Eppendorf tubes and ultra-pure (18.2 Ω) H_2_O were transferred into new tubes for extraction; this ensured that all samples had the same RI.

### Strategy for Selection of Candidate Dietary Exposure Biomarkers

The selection of biomarkers was initiated with a literature search to generate an initial “long list” of food-related metabolites with potential for inclusion in a panel of biomarkers that would provide comprehensive coverage of food items consumed in the MAIN Study (see Supporting Data 2 for a summary of the foods**)**. The search was carried out using Google Scholar and Web of Knowledge using the following search terms in a range of combinations “biomarkers + urine + food + dietary + BFI” and ended on 22/06/2020. Publications were screened and information was added to the database if they contained data relating to potential dietary exposure biomarkers measured in urine samples (see Supporting Data 3). Specific details on metabolite excretion profile were recorded and the availability of a commercial supply of a pure chemical standard was investigated (see Supporting Data 3).

### Evaluation of Chemical Diversity of Biomarkers

Biomarkers were assigned to chemical class and superclass using the ClassyFire application (71). Classifications for each biomarker were retrieved using the R package classyfireR Version 0.3.3. Pairwise similarity of biomarkers was measured using the Tanimoto Distance after converting structural representations of each biomarker to its MACCS (Molecular ACCess System) fingerprint. Fingerprints were generated using the get.fingerprint function from the R package rcdk (Version 3.5) and distances computed using the fp.sim.matrix function from the R package fingerprint. The resultant matrix of fingerprint distances, was then reduced to two dimensions using the cmdscale function. Chemical descriptors (–log*P*) were calculated using the rcdk package.

### Sample Analysis by Liquid Chromatography Triple Quadrupole Mass Spectrometry (LC-QQQ-MS)

Methanol (primer trace analysis grade, Fisher Scientific, UK) was used for urine extraction and standards preparation. Acetonitrile (Optima® LC-MS grade, Fisher Scientific, UK), methanol (HPLC grade, Fisher Scientific, UK), and Ammonium acetate (Optima® LC-MS grade, Fisher Scientific, Belgium) were used for preparing the LC mobile phase. Water was ultra-pure water (18.2 Ω) drawn from an Elga Purelab® flex water purifier system (Taiwan). The suppliers of chemical standards are given in Supporting Data 4.

Sample analysis was performed on a TSQ Quantum Ultra EMR QQQ mass spectrometer (Thermo Scientific) equipped with a heated electrospray ionisation (HESI) source. Samples were delivered using an Accela ultra-high-performance liquid chromatography (UHPLC) system (Thermo Scientific) consisting of autosampler, column heater, and quaternary UHPLC-pump. For HILIC (Hydrophilic Interaction Liquid Chromatography) analysis, chromatographic separation was performed on a ZIC-pHILIC (polymeric 5 μm, 150 × 4.6 mm) column (Merck). The mobile phase consisted of 10 mM ammonium acetate in water: acetonitrile (95:5) (**A**) and 10 mM ammonium acetate in water: acetonitrile (5:95) (**B**). The gradient program used was as follows: 0 min, 95% B (400 μL min^−1^); 15 min, 20% B (400 μL min^−1^); 15.01 min, 20% B (500 μL min^−1^); 20 min, 20 % B (500 μL min^−1^); 20.01 min, 95 % B (500 μL min^−1^); 25 min, 95% B (500 μL min^−1^). The HPLC was carried out in low pressure (~0–7,000 psi) operating mode with 0 psi and 650 psi as minimum and maximum pressures, respectively. For Reverse Phase (RP) analysis, chromatographic separation was performed on a Hypersil Gold (1.9 μm, 200 × 2.1 mm) RP-column (Thermo Scientific). The mobile phase consisted of 0.1% formic acid in H_2_O (**A**) and 0.1% formic acid in MeOH (**B**). The gradient program used was as follows: 0 min, 0% B; 0.5 min, 0% B; 5 min, 60% B; 11 min, 100% B; 13 min, 100% B; 13.01 min, 0% B; 19 min, 0% B. For RP analysis, the flow rate was maintained at 400 μL/min^−1^. The UHPLC was carried out in high pressure (~7,000–15,000 psi) operating mode with 0 and 1,000 psi as minimum and maximum pressures, respectively. For both chromatographic analyses, column oven and autosampler tray were maintained at 60 and 14°C, respectively. To ensure consistent sample delivery, 20 μL were injected using a 20 μL loop and partial loop injection mode. After each injection, syringe, and injector were cleaned using a 10 % HPLC grade MeOH solution in ultra-pure water (1 mL flush volume; 100 μL/s^−1^ flush speed, 2 mL wash volume) to avoid sample carryover. Mass spectra were acquired in multiple reaction monitoring (MRM) mode, in positive and negative ionisation polarities simultaneously using optimised values of collision energy and tube lens for each MRM transition (Supporting Data 4). Spectra were collected at a scan speed of 0.010 and 0.003 s for HILIC and RP analysis, respectively. A scan width of 0.010 m/z units and peak width (Q1, Q3) of 0.7 FWHM were used for both HILIC and RP analyses.

Raw files (ThermoFisher) were converted to mzML (72) using msconvert in the ProteoWizard tool kit (73). All further processing of mzML files was performed using the R Statistical Programming Language (74). Selected Reaction Monitoring (SRM) chromatograms were extracted from mzML files using the R library, mzR and peaks areas were calculated by extracting pre-defined chromatographic windows (based on calibration standards) around each peak apex. Absolute concentrations were calculated using a nine-point calibration curve (0.006561–100 μg mL^−1^). The limit of detection (LoD) and limit of quantification (LoQ) of all chemical standards were calculated as the lowest concentration of each biomarker giving a signal-to-noise ratio of 3:1 and 10:1, respectively within the linear range of each calibration curve.

### Quality Control (QC) Strategy for Target Biomarkers

Reproducibility of the mixture of chemical standards was determined using the relative standard deviation (RSD) of a multi component calibration standard and an external urine QC sample using a “master mix” of pooled samples. The external urine QC sample was used to determine the effect of the resultant urine matrix on the reproducibility of selected biomarkers across multiple experiments. The external QC (as distinct from an experimental QC) allowed for longitudinal monitoring of RSD without intra-experimental bias.

### Data Analysis

Principal Components Analysis (PCA) was performed using the *prcomp* function in R, with variables scaled to unit variance. Supervised classification of quantitative metabolite data was performed using Random Forest (RF) classification using the randomForest package (75) in R (74). For all RF models, the number of trees (*ntree)* used was 500 and the number of variables considered at each internal node (*mtry*) was the square root of the total number of variables. Accuracy, margins of classification and area under the ROC (Receiver Operator Characteristic) curve (AUC) were all used to evaluate the performance of classification models, as described previously (76). RF classification models were plotted following multi-dimensional scaling (MDS). Proximity measures for each individual observation were extracted from RF models and scaled coordinates produced using cmdscale on *1—proximity*.

Spearman rank correlations of biomarker concentrations in 24 h vs. FMV urine were produced using the *rcorr* function from the R (Version 4.0.3) package Hmisc (Version 4.4.0). Reported *P*-values are the asymptotic *p*-values from the rank correlation. Quantile–Quantile plots were produced using the *qqnorm* function in R.

## Results

### Selection of Target Foods and Design of a Food Intervention Study for Preliminary Survey of the Potential Utility of Urine Biomarker Technology

A major component of our strategy to develop urine biomarker technology to monitor habitual diet was the need for a biobank of urine samples from a food intervention trial that was designed to provide comprehensive exposure to foods commonly consumed in the UK. Key food groups were identified initially from *The Eatwell Guide* (77); the most commonly eaten foods were identified within each disaggregated food group using estimates of intakes of foods by the UK population from the UK National Diet and Nutrition Survey (NDNS) (21). Supporting Data 2 describes the representative foods that were incorporated into a six-menu design as part of the MAIN food intervention trial at Newcastle [see Lloyd et al. (1) and Willis et al. (2) for full details]. The menu plans aimed to deliver foods for BFI discovery and validation, including the assessment of BFI specificity and sensitivity within the context of the whole diet. Particularly important was consideration of the impact of the likely major sources of variance on biomarker monitoring procedures including:

- The impact of exposure to targeted foods as part of complex and mixed meals, rather than foods consumed in isolation;
- The use of average portion sizes and normal eating patterns rather than exposure to huge, unrealistic portions consumed in a fasted state;
- The impact of different food formulations, processing, and cooking methods representing the range of ways in which foods are processed and eaten;
- The dynamics of putative BFI retention in the body (to inform development of biomarkers of both acute or habitual food consumption).

The selection of biomarkers was initiated with a preliminary literature search to identify putative urinary biomarkers that would provide comprehensive coverage of each specific food/food group consumed within the six menus. This database (Supporting Data 1) suggested that 765 urinary metabolites were potential dietary exposure biomarker candidates as summarised in Supporting Data 2. It is clear from these data that there is considerable choice in terms of potential biomarkers and considerable overlap of metabolites between some foods/food groups.

### Evaluation of Approaches for Urine Collection and Storage for Monitoring Habitual Dietary Exposure

**Figure 2:**
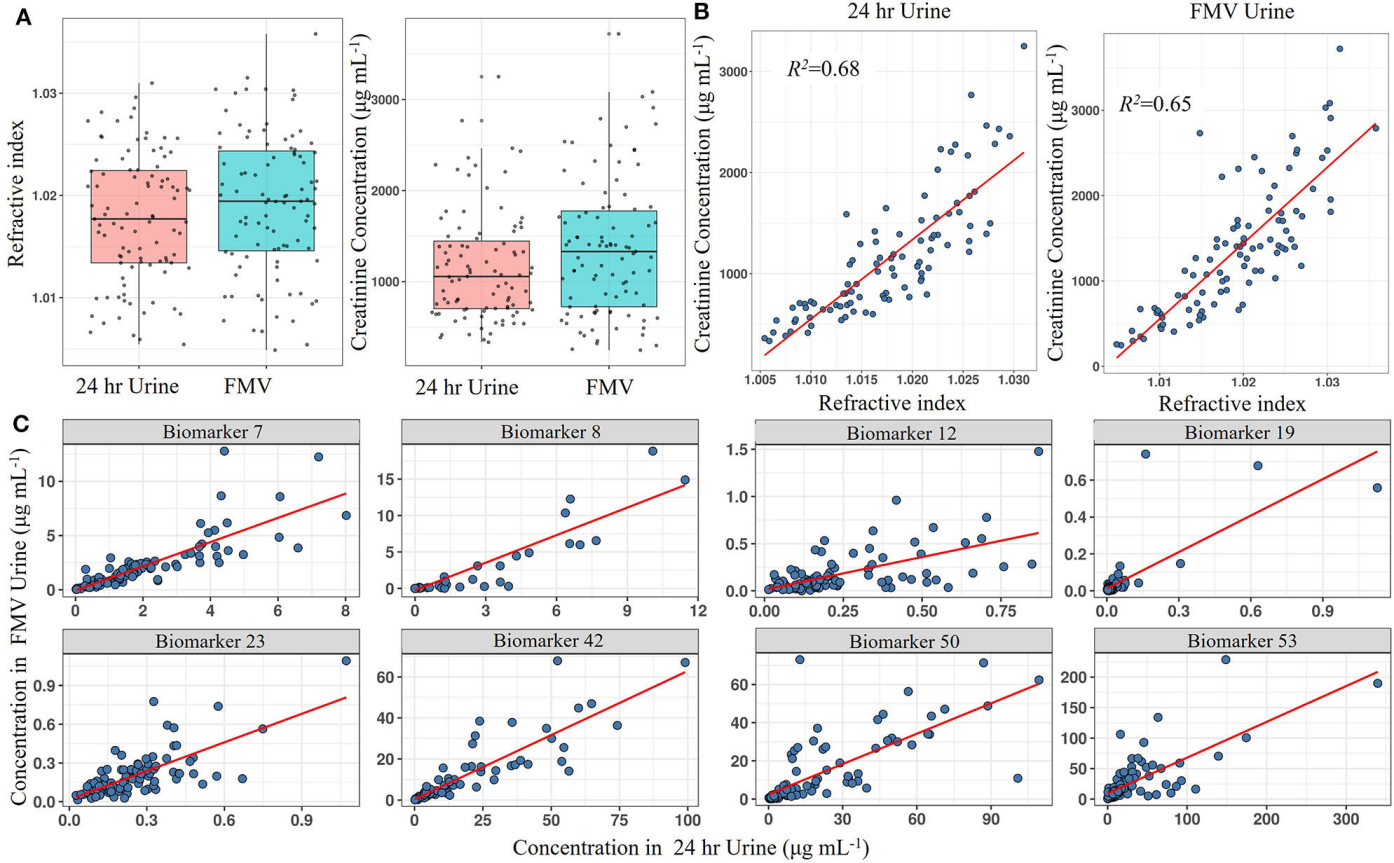
Screening biomarkers to detect those with concentrations in spot urine that reflect levels found in 24 h urine from ***Study 1***. **(A)** Boxplot of total creatinine content and refractive index (RI) in First Morning Void (FMV) and 24 h urine samples. **(B)** Scatter plot showing the linear association between creatinine concentration and RI in FMV and 24 h urine samples. **(C)** Scatter plots showing the linear association of selected biomarker concentrations between FMV and 24 h urine samples. Where: Biomarker 7,7-Methyl xanthine; Biomarker 8, Acesulphame-K; Biomarker 12, Calystegine A3; Biomarker 19, D,L-Sulphoraphane-N-acetyl-L-cysteine; Biomarker 23, DHPPA [3-(3,5-Dihydroxyphenyl)-1-propanoic acid]; Biomarker 42, Proline betaine; Biomarker 50, Tartarate; Biomarker 53, Trimethylamine-N-oxide (Full list of number codes for biomarkers is in Supporting Data 4).

Using LC-MS fingerprinting methodology, we have shown previously that the metabolome of spot urine samples taken just before bed time on the study day is compositionally very similar to the corresponding 24 h urine samples (62). In the present study, RI measurements revealed that FMV spot urine samples from a national dietary intake survey (***Study 1***) also had an almost identical overall solute concentration range to that of 24 h urines (Figure 2A). Since creatinine concentrations are often used as a reference for normalisation in urine samples, we evaluated the relationship between creatinine concentration and RI in both FMV spot and 24 h urines. Creatinine concentrations were within the same range in FMV spot and 24 h urines and exhibited a strong linear relationship (*R*^2^ = 0.65 and 0.68, respectively) with RI (Figure 2B), supporting the concept of sample normalisation to the same RI. The value of FMV spot urine samples for assessment of dietary exposure was explored further by examining the correspondence between the concentration of putative biomarkers in 24 h urine and spot urine samples using targeted, quantitative measurements of individual biomarkers. Figure 2C shows scatter plots of metabolite concentration in FMV urine vs. 24 h urine for eight example biomarkers from samples derived from ***Study*** ***1***. Although the actual biomarker levels varied between the two urine types there was a strong linear relationship between concentrations in 24 h and FMV urine (Figure 2C). More than 50% of the biomarkers demonstrated a very strong correlation (>0.6). Further potential biomarkers exhibited a weaker correlation in concentration (Supporting Data 4) and it is suggested that that *r* > 0.2 (with a *p* < 0.05 from a rank correlation test) may be considered adequate. The Quantile–Quantile plot in Supporting Data 5 shows the comparable distribution of biomarker concentrations measured in FMV and 24 h urine samples.

**Figure 3:**
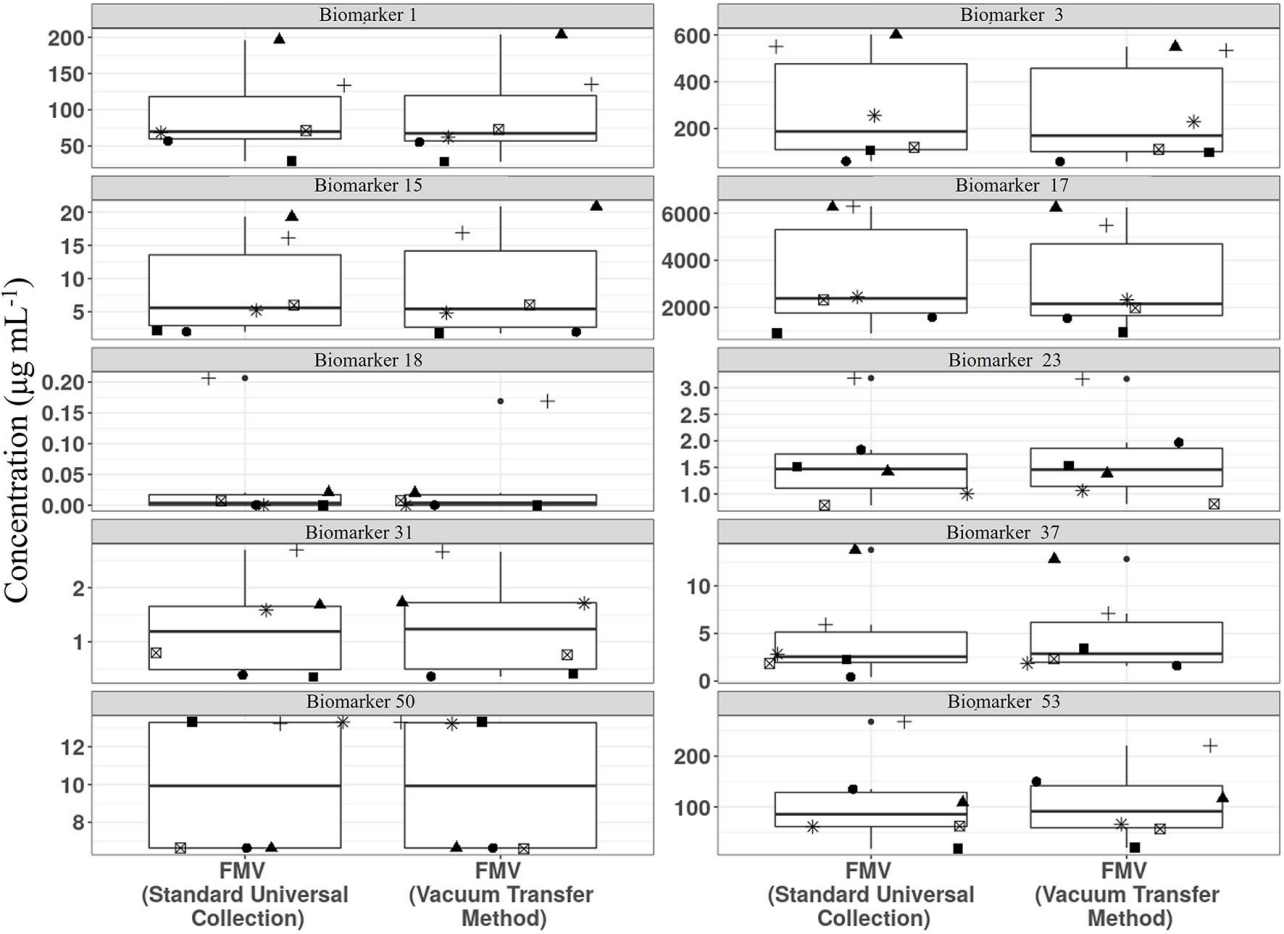
Comparison of stability of example biomarkers in First Morning Void (FMV) urine collected in vacuum tubes stored at 4°C for 2 weeks (Vacuum transfer method) or Universal tubes (Standard Universal collection) stored at −80°C. Where: Biomarker 1, 1-Methyl histidine; Biomarker 3, 3-Methyl histidine; Biomarker 15, Carnosine; Biomarker 17, Creatinine; Biomarker 18, D,L-Sulphoraphane L-cysteine; Biomarker 23, DHPPA [3-(3,5-Dihydroxyphenyl)-1-propanoic acid]; Biomarker 31, Ferulic acid-4-O-sulphate; Biomarker 37, L-Anserine; Biomarker 50, Tartarate; Biomarker 53, Trimethylamine-N-oxide (Full list of number codes for biomarkers is in Supporting Data 4).

We have shown recently that vacuum tube technology has considerable value for spot urine sampling and that, even in the absence of preservatives, urine composition is stable for several days at 4°C (67) and under different temperature regimes. To explore further the utility of vacuum tube technology for large-scale urine sampling in community settings, we evaluated the compositional stability of FMV spot urine at 4°C for 2 weeks, to mimic longer term storage in a domestic fridge. A selection of biomarkers useful for assessment of exposure to meat, fish, wholegrain, fruit, and vegetable components of meals were targeted for analysis. Metabolite concentrations after storage in vacuum tubes at 4°C were very similar to those of the same urine samples after being frozen at −80°C (Figure 3).

### Literature Analysis to Select Biomarker Leads for Inclusion in a Panel That Will Provide a Comprehensive Survey of Dietary Exposure

A comprehensive list of potential urinary BFIs based on a literature analysis (53) of putative dietary exposure biomarkers in various human biofluids is presented in Supporting Data 2. The present biomarker panel strategy aimed to assess habitual diet in individuals and populations; key to this objective was the need to use spot urine samples, specifically urine samples collected just before bedtime and FMV urines, that would be informative of overall food consumption (1, 62). A detailed examination of the dietary exposure biomarker literature was undertaken with particular emphasis on the identification of biomarker candidates present in spot urine samples > 12 h after food consumption (Table 2 and described in further detail in Supporting Data 3). A shortlist of candidate biomarkers for initial biomarker panel development was generated, focusing largely on metabolites that were available from commercial providers (Supporting Data 3). For 28 out 54 putative biomarkers there was already evidence in the literature of their presence in FMV urine. The majority of the remaining dietary exposure biomarker leads selected had been shown to be present in 24 h urine samples and so it was reasonable to expect their presence in FMV urine samples collected the day after a specific food intervention.

**Table 2:** Selection of biomarkers for panel development.

**Dietary component**	**Putative urine biomarker**	**Potential use as a habitual dietary exposure biomarker?**	**Key reference in relation to habitual dietary exposure biomarker potential**
Alcohol	Ethyl-beta-D-glucuronide	**	(78)
Wine	Resveratrol	*	(59)
Coffee	Chlorogenic acid	*	(79)
Coffee	Dihydrocaffeic acid^#^	*	(80)
Coffee	Ferulic acid-4-O-sulphate	*	(80)
Coffee	Feruloylglycine	**	(1)
Coffee	m-Coumaric acid^#^	*	(46)
Cocoa	3-Methyl-xanthine	**	(81)
Cocoa	7-Methyl-xanthine	**	(81)
Cocoa/Tea	Caffeic acid^#^	*	(80)
Coffee/Cocoa	Caffeine	*	(81)
Cocoa	Vanillic acid	**	(46)
Sweetener	Acesulphame-K	**	(2)
Sugary Foods and Drinks	Sucrose	**	(39)
Fruit and Vegetables	3-Hydroxyhippuric acid	**	(82)
Fruit and Vegetables	4-Hydroxyhippuric acid	**	(82)
Fruit and Vegetables	Hippuric acid	*	(83)
Citrus	4-Hydroxyproline-betaine	**	(30)
Banana	Dopamine-3-O-sulphate^#^	**	(84)
Banana	Dopamine-4-O-sulphate^#^	**	(84)
Strawberries/red berries	Furaneol	**	(2)
Citrus (grapefruit)	Naringenin	*	(85)
Grapes/wine/red berries	p-Coumaric acid	**	(86)
Citrus	Proline betaine	**	(30)
Apple	Rhamnitol	**	(22)
Grapes	Tartarate	**	(87)
Onion and tomato	Quercetin	*	(11)
Onion and tomato	Quercetin-3-O-b-D-glucuronide	**	(88)
Cruciferous Vegetables	D,L-Sulphoraphane L-cysteine	**	(89)
Cruciferous Vegetables	D,L-Sulphoraphane-N-acetyl-L-cysteine	**	(1)
Wholegrain/Rye	BOA (1,3-Benzoxazol-2-one)	*	(35)
Wholegrain	DHBA (3,5-Dihydroxybenzoic acid)	*	(90)
Wholegrain	DHBA-3-O-sulphate	*	(91)
Wholegrain	DHPPA (3-(3,5-Dihydroxyphenyl)-1-propanoic acid)	*	(90)
Wholegrain	DHPPA-3-sulphate	*	(91)
Meat (general)	1-Methyl histidine	*	(92)
Poultry/Fish	3-Methyl histidine	*	(27)
Meat (processed)	Carnitine	*	(93)
Red meat	Carnosine	*	(24)
Meat (general)	Creatinine	*	(25)
Chicken	L-Anserine	**	(1)
Meat (general)	Taurine	*	(25)
Fish/Shellfish	Trimethylamine-N-oxide	**	(1)
Potatoes	Calystegine A_3_	**	(48)
Potatoes	Calystegine B_2_/B_1_	**	(48)
Soy products	Daidzein	**	(94)
Legumes	Pyrogallol	**	(2)
Legumes	Trigonelline	**	(2)
Strongly Heated Foods	N-(2-Furoyl)glycine	**	(2)
Polyphenol rich foods	Epicatechin(-)	*	(79)
Polyphenol rich foods	Ferulic acid	*	(95)
Polyphenol/Anthocyanin rich foods	Ferulic acid-4-O-b-D-glucuronide	*	(96)
Fruit/Grapes/Tea/wine	Gallic acid	*	(86)
Anthocyanin rich foods	Protocatechuic acid	**	(97)

# Normally the conjugated forms detected; the impact/use of the selected metabolite as a potential biomarker of habitual dietary exposure where *Possible and **Likely.

### Examination of Biomarker Behaviour During LC-MS and Development of a Biomarker Panel Strategy

**Figure 4:**
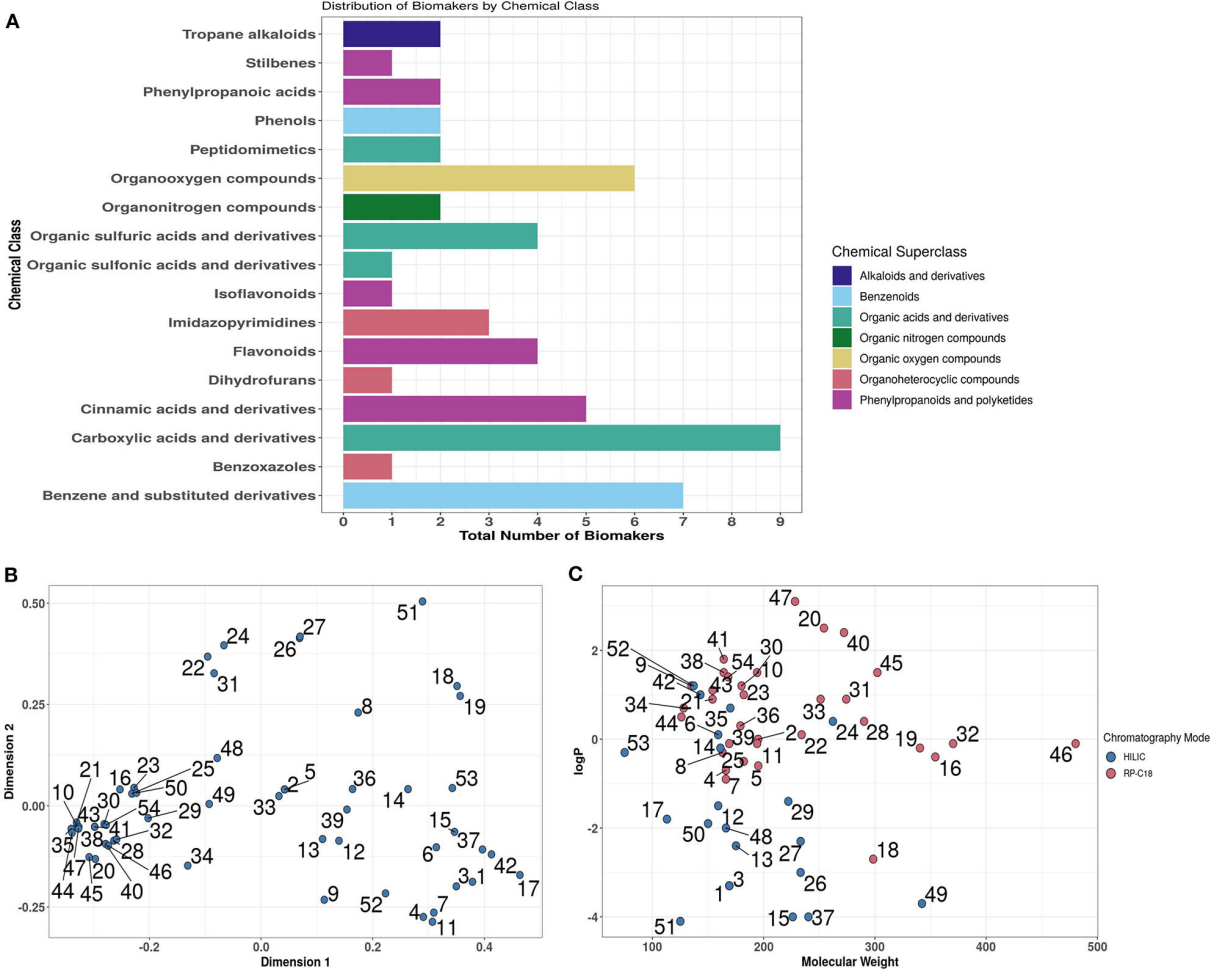
*In silico* overview of the chemical diversity of candidate biomarkers. **(A)** Chemical class and Superclass classifications of 54 biomarkers using ClassyFire. **(B)** Multi-dimensional scaling of Tanimoto Distances between MACCS (Molecular ACCess System) fingerprints of biomarkers. **(C)**. Visualisation of biomarkers in chemical space. Where: log*P*, partition coefficients [Full list of number codes for biomarkers in panels **(B,C)** is in Supporting Data 4].

Previous analysis of published literature revealed that the great majority of dietary exposure biomarker candidates were detected and quantified using LC-MS technology (53). Chemical classification of putative biomarkers showed great structural diversity that included metabolites from 17 Chemical Classes representative of 7 Chemical Super-Classes (Figure 4A). *In silico* multi-dimensional scaling of structural attribute fingerprint distances shows the large diversity in chemical structure across biomarker candidates, highlighting the necessity for employing multiple chromatography systems (Figure 4B). Focusing specifically on partition coefficients (log*P*) and molecular weight attributes, it is clear from the scatterplot shown in Figure 4C that a large percentage of biomarker candidates were quite strongly hydrophilic. Based on this distribution a decision was made to develop an analytical strategy based largely on the use of a HILIC column to measure strongly polar chemicals and a RP (C_18_) column to quantify less polar metabolites.

**Figure 5:**
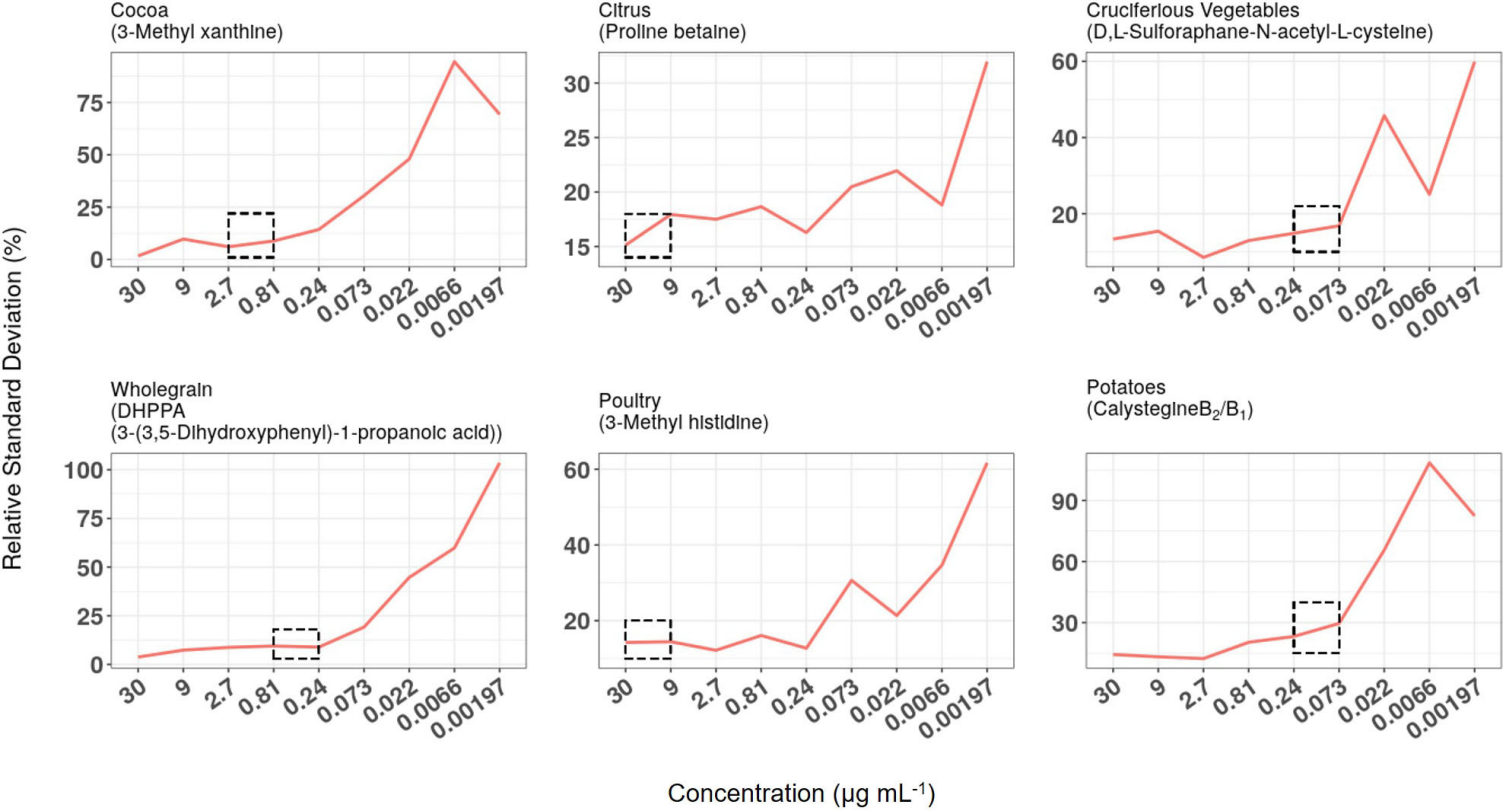
Relationship between relative standard deviation (RSD), biomarker concentration in calibration standard mixtures, and median concentration in First Morning Void (FMV) urines from ***Study 1***. RSD data of biomarkers used to monitor exposure to six example foods/food groups when measured at nine concentration levels over a 6-month period. The concentration range within which the median concentration of the same biomarkers was measured in FMV urine taken from 95 free-living participants are highlighted by dotted boxes.

LC-QQQ-MS/MS technology is used widely for measuring, with high sensitivity, the concentration of target chemicals in complex biological samples. Quantification of pre-determined fragmentation products of targeted metabolites in expected retention time windows using MRM approaches allows the investigator to obtain data on large numbers of individual metabolites in short (10–15 min) HPLC runs. Chemical mixtures designed as calibration standards for either HILIC or RP (C_18_) chromatography were used to optimise metabolite separation and detection conditions on a Thermo QQQ instrument (see Supporting Data 4). Serial dilutions of the two standard chemical mixtures (30–0.00197 μg ml^−1^) were used to establish LoD and LoQ and to examine analytical reproducibility over several months. The reproducibility of measurement of chemical standard mixtures was determined at nine concentration levels. RSD data for biomarkers used to monitor exposure to six example foods/food groups are illustrated in Figure 5 and in all cases, reproducibility gradually worsened as biomarker concentration dropped. Median concentrations of the same biomarkers were measured in FMV urine taken from 95 free-living participants from ***Study 1***. For each metabolite the median concentration was substantially greater than the level at which RSD approached 20% (see dotted boxes in Figure 5) and usually an order of magnitude greater than the LoQ (see Supporting Data 4).

### Demonstration of Biomarker Panel Utility to Examine Eating Behaviour in the MAIN Study

**Figure 6:**
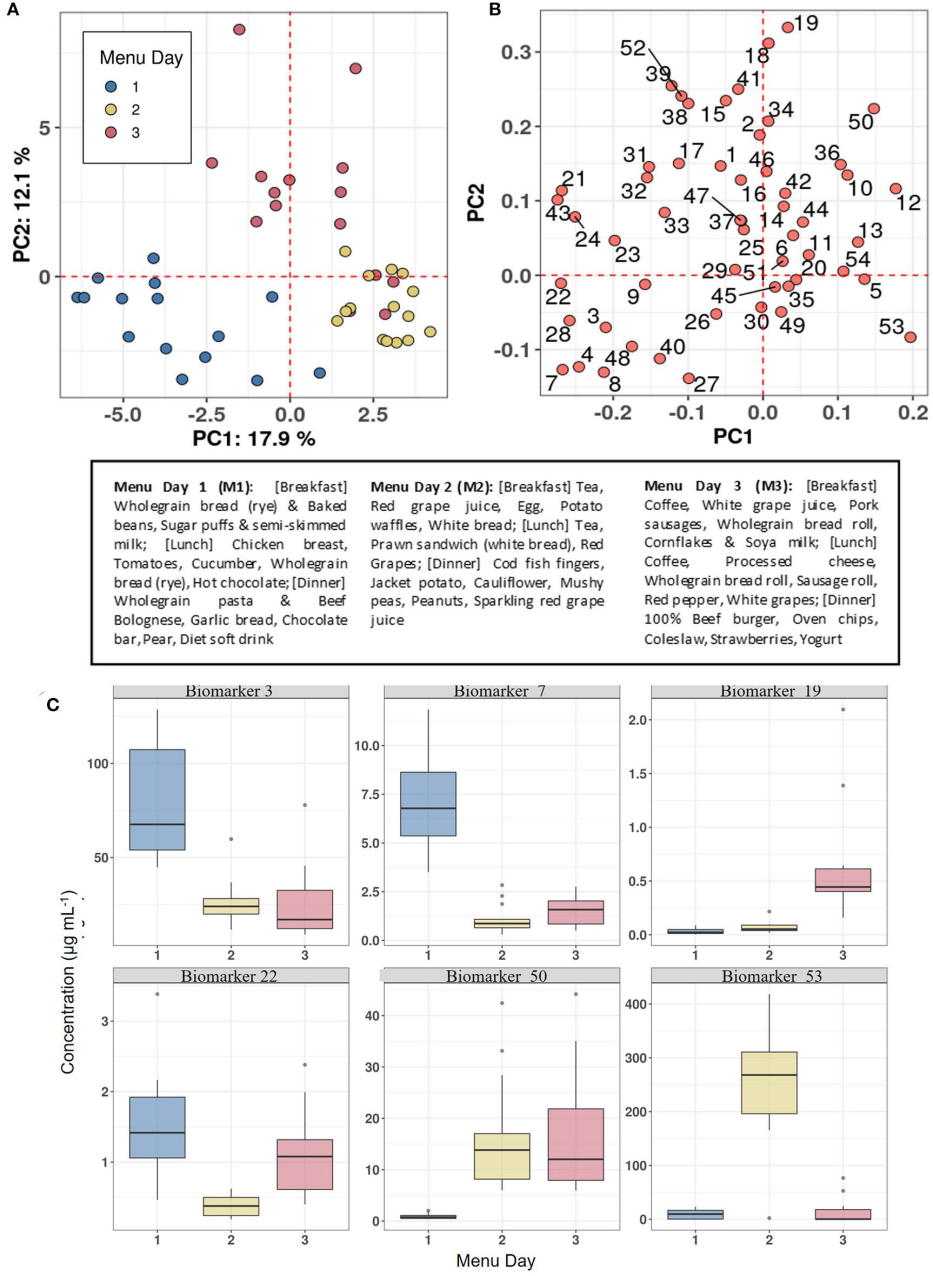
Biomarker panel to characterise eating behaviour on individual Menu Days in ***Study 2***. **(A)** Principal Components Analysis (PCA) scores plot of biomarker panel measurements in First Morning Void (FMV) urine of 15 individuals across three Menu Days in ***Study 2***. **(B)** PCA variable loadings plot showing the variance contributions of biomarkers on each Menu Day (Full list of number codes for biomarkers in panel B is in Supporting Data 4). **(C)** Boxplots illustrating the concentration in FMV urine of top ranked biomarkers discriminating Menu Days following Random Forest classification. Text box provide details of meals consumed on each Menu Day. Where: Biomarker 3,3-Methyl histidine; Biomarker 7,7-Methyl xanthine; Biomarker 19, D,L-Sulphoraphane-N-acetyl-L-cysteine; Biomarker 22, DHBA-3-O-sulphate; Biomarker 50, Tartarate; Biomarker 53, Trimethylamine-N-oxide.

The utility of the biomarker panel to characterise eating habits within populations was explored by measuring the concentrations of 54 BFIs in FMV urine samples obtained on days following consumption of three distinctive meal plans (see text box in Figure 6) from 15 individuals in the Newcastle MAIN food intervention study (***Study 2***). The data were subjected to PCA which showed distinctive clustering of urine samples by Menu Day (colour coded) in relation to the zero position in PC1 and PC2 (indicated by dotted red grid lines in the scores plot shown in Figure 6A). Menu Day 1 and Menu Day 2 samples separated strongly in PC1, whereas Menu Day 3 samples clustered away from samples representative of the other 2 Menu Days in the PC2 dimension. Biomarkers that are strongly explanatory of differences in the composition of urines collected the morning after individual Menus Days are shown in Figure 6B. Examination of the 4 sectors delineated by the zero grid lines of the loadings plot revealed a strong association between specific biomarkers and particular foods consumed on each menu day. For example, TMAO (60) was strongly associated with cod fish fingers consumed on Menu Day 2, 3-Methyl histidine (3) was linked to chicken consumption on Menu Day 1 and carnosine (15) was indicative of exposure to a 100% beef burger on Menu Day 3. 1- and 3-Methyl-xanthine (4 and 7) and Epicatechin(–) (28; a marker for general polyphenol-rich foods) detected exposure to cocoa products on Menu Day 1, whilst Acesulphame-K (8) was associated with exposure to a diet soft drink on the same day. Sulphoraphane derivatives (18 and 19; D,L-Sulphoraphane L-cysteine and D,L-Sulphoraphane-N-acetyl-L-cysteine) were highly explanatory of exposure to coleslaw (containing cabbage) on Menu Day 3, whilst trigonelline (53) and N-(2-Furoyl)glycine (39; a strongly heated food marker) reflected exposure to coffee on the same day. Tartrate (51) and the calystegines (12 and 13; A_3_ and B_2_/B_1_) contributed strongly to the clustering of urine samples from Menu Days 2 and 3 away from Menu Day 1 samples when grape products and potato products were not consumed. The example box plots (colour coded by Menu day) in Figure 6C demonstrate a clear increase in the concentration in urine of the selected biomarkers the day after the consumption of a specific food.

**Figure 7:**
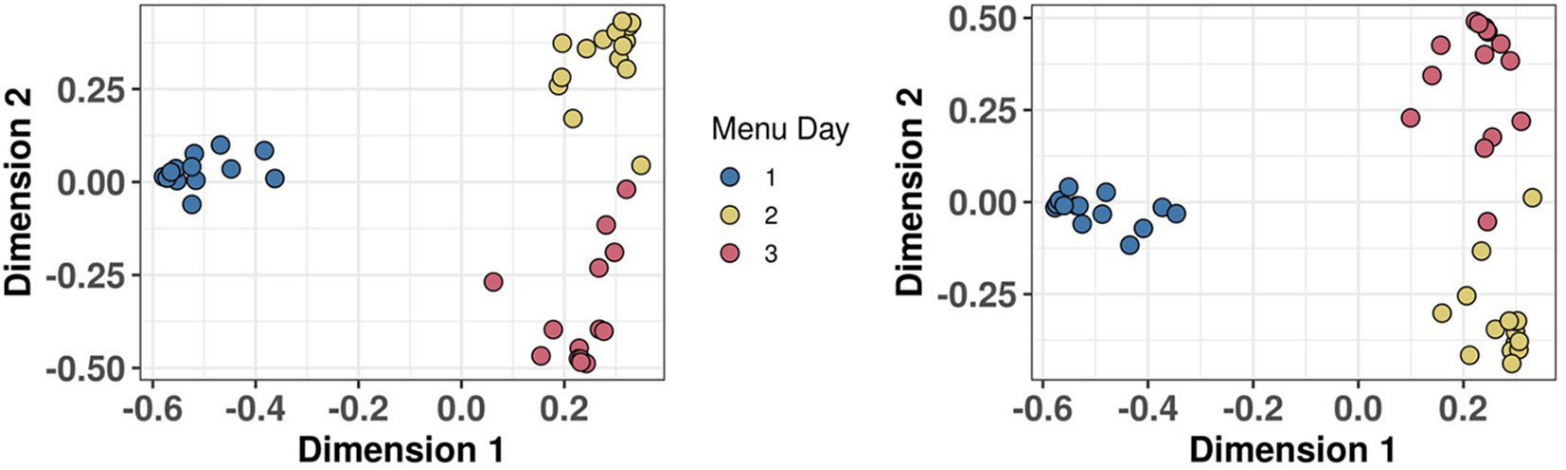
Demonstration that biomarker panel can be extended without any loss of classification power. Multi-dimensional scaling (MDS) of Random Forest proximity extracted from a classification model of 15 individuals consuming three unique menus in ***Study 2***. **(A)** MDS using a panel of 38 biomarkers of food intake (BFIs) which was extended to 54 BFIs and **(B)** 2 years later.

An important feature of any biomarker strategy designed to monitor habitual diet in both individuals and populations is the ability to add in new biomarkers as they are discovered and validated. RF can be used to assess the stringency of sample classification based on modelling output measures such as accuracy, AUC, and margins (76). Figure 7 shows a MDS of proximity scores extracted from a RF classification model of the same 15 individuals consuming three unique menus, and panels of 38 dietary biomarkers used in 2018 (Figure 7A) and extended to 54 biomarkers in 2020 (Figure 7B). In both models, sample clustering by Menu Day is very similar and modelling output measures are still excellent, despite the challenge of measuring many more biomarkers in each MRM experiment in the more complex panel utilising 54 biomarkers.

## Discussion

More than a decade of intensive research to discover putative BFIs has yielded a wealth of information highlighting specific metabolites that appear in urine following consumption of individual foods/food groups. A large number of such metabolites have great potential as biomarker leads for particular foods/food groups when validated in isolation (49). However, their deployment in any cost-effective strategy aimed at comprehensive monitoring of habitual diet imposes a substantial number of further challenges that require both definition and investigation (1, 22). Preliminary investigation of the performance of any specific biomarker in the context of a complex biomarker panel requires urine samples from complex food intervention studies designed specifically to emulate habitual eating patterns. The MAIN Study at Newcastle was designed with this specific objective in mind (1, 2, 22). By validating biomarkers in urine samples from studies researching eating behaviour in free-living populations (62, 67) it is anticipated that BFI technology will mature rapidly over the next few years.

It is particularly important to consider carefully urine sampling approaches when deploying BFI technology to help monitor diet and to adopt a methodology that is appropriate for the study objectives (see Table 1). Twenty-four hour urine samples, which include the FMV after the study day, provide an ideal type of sample to assess food intake on a single day with the caveat that their collection imposes considerable burden on study participants. To assess habitual diet using BFI data would demand the collection 24 h urine samples on multiple days which can have a substantial influence on the acceptability of the study requirements and compliance by participants, as well as impacting greatly on study logistics and overall costs (1, 62, 67). Consequently, spot urine samples are becoming the urine samples of choice for studying BFIs of a single food/food group when compared with 24 h collections [e.g., (89, 98)] because their collection has little impact on normal daily activities of study participants.

Nutrikinetic studies of potential BFIs have shown that diet-derived metabolites from individual foods reach peak levels in urine at different times post-prandially (49). Thus, choosing an appropriately-timed spot urine sample is clearly problematical when considering the effective deployment of a comprehensive biomarker panel covering the whole of diet. Our recent studies have shown that spot urine samples are generally adequate substitutes for 24 h urine samples for measurement of BFIs (22, 62); particularly post-evening meal (i.e., just before bed time) and FMV urines were collected with a high degree of success (22). In the present paper, we describe a strategy to select urinary biomarkers for inclusion in a comprehensive and extendable panel to monitor habitual dietary exposure that focuses on the use of FMV urine samples. From an analytical perspective, sampling FMV urines after a substantial overnight sleep period allows sufficient time to elapse for any gut microbiome and liver P450 bio-transformations of targeted metabolites to be completed, thus extending the availability of characteristic biomarkers and increasing their concentrations in the collected urine. It has been shown previously that the distributions of biomarker concentrations are comparable between post-dinner spot samples, overnight cumulative samples, and 24 h urines (62). We demonstrated recently (53) that more than 50 different potential BFIs were detectable in FMV urine the day after the consumption of targeted foods. In the present study, we show that for many, but not all, BFIs there is a relatively linear correlation between concentration in 24 h and FMV urine and suggest that only those with an *R*^2^ approaching 0.2 may be suitable for accurate quantification when using a comprehensive biomarker panel to monitor habitual diet. Although this is clearly a limitation for accurate quantification of food intake it is very likely that the presence in FMV urine of BFIs with lower correlation coefficients will still provide a useful qualitative indication of recent exposure to their target foods.

Our recent collaborations have highlighted the importance of understanding metabolic biotypes (metabotypes) in populations that may impact on nutritional status (41, 45, 99). As many dietary exposure biomarkers are derived from food chemicals that are metabolised and/or biotransformed before excretion, it is possible that chemical “signatures” reflective of common metabotype groupings in any population can be visualised using a biomarker panel. Differential metabolism of any particular BFI by metabotype sub-groups in any population would provide an additional limitation on its utility for quantitative assessment of dietary intake. The hydration levels of study participants can vary considerably and has to be adjusted for in any BFI deployment strategy. The use of 24 h urine samples for biomarker quantification demands the accurate measurement of the total volume of urine produced during any 24 h period and then the concentration and extraction of a specific aliquot before analysis in order to calculate the overall daily excretion rate.

Logistics, the analytical and computational skills required, and costs will also impact on the wider acceptance and adoption of dietary exposure biomarker technology by the nutrition research community. In the methodology we describe, urine processing is limited to a simple dilution with ultra-pure water as QQQ instruments operating in MRM mode are extremely sensitive and thus there is a need to collect only small volumes of urine for analysis (e.g., 0.5–3 ml). With this objective in mind, we have shown that spot samples can be collected in the home with high collection compliance using vacuum tube technology (67). Importantly, urine samples collected by this method are compositionally stable at room temperature for several days without preservatives (67). This feature of vacuum tube collection methodology allows transport by domestic mail without dry ice offering the opportunity to scale up dietary exposure studies in community settings. A commercial product for spot urine collection (Supporting data 6) is now on the market (https://www.co-vertec.co.uk/) and is currently under evaluation in several clinical trials interested in monitoring vulnerable populations in community environments to study malnutrition (https://www.hra.nhs.uk/planning-and-improving-research/application-summaries/research-summaries/stream-feasibility-study/), impact of homelessness on diet (100) and evaluating the eating behaviours in pre-diabetic individuals (https://waru.org.uk/cms/waru_news/targeting-pre-diabetes-through-primary-care/).

Targeted profiling of urine using high resolution hybrid quadrupole/ion trap technology, coupled with RP C_18_ UHPLC, can capture information about the relative concentrations of a substantial number of metabolites in a sample when combined with urine concentration by solid phase extraction methodology [e.g., (101)]. However, sample processing can add significant time and cost to any analytical process and in our experience differential metabolite recovery from ion exchange cartridges can add a significant degree of uncertainty and variance in metabolite measurement. In the present study, we have described a fully quantitative approach using QQQ-MS/MS to measure biomarker abundance. This methodology uses complex mixtures of chemicals standards for quantitation and utilises two HPLC columns solutions to provide optimal resolution of a structurally diverse range of chemicals using short chromatography runs.

As outlined in Table 1 the utility of any biomarker panel will depend on the study objectives. The biomarker panel described in the present study was optimised specifically to investigate eating behaviour in free-living populations and was targeted towards frequently consumed foods of high public health importance in the UK (2). One limitation of the present study is that only relatively small populations have been used in these initial validation studies and in future BFI technology will need to be tested rigorously in multiple larger populations. We have shown that the biomarker panel can be extended incrementally as new biomarker leads are evaluated and current evidence suggests that it should be straightforward to adapt our strategy to develop biomarker panels that provide comprehensive coverage of foods consumed frequently in other populations. Combined with existing bespoke software for data extraction, it is expected that the development of high throughput, automated biomarker measurement procedures to assess dietary intake is within scope in the near future. In addition, the routine generation of quantitative BFI data will offer further opportunities to develop novel “healthy eating indices” to summarise and “score” eating habits for use in personalised nutrition applications (26, 45). In conclusion, we believe that the integration of information from BFI technology and dietary self-reporting tools, combined with a deeper understanding of nutritional metabolic biotypes in populations, will help to provide more robust understanding of the complex interactions between dietary behaviour and human health.

## Data Availability Statement

The raw data supporting the conclusions of this article will be made available by the authors, without undue reservation.

## Ethics Statement

Study 1 involving human participants were reviewed and approved by the National Commission for Data Protection, the Ethical Committee of the Institute of Public Health of the University of Porto and from the Ethical Commissions of each one of the Regional Administrations of Health. All participants gave written informed consent, and the study was carried out in accordance with the Declaration of Helsinki. Study 2 was reviewed and approved by East Midlands—Nottingham 1 National Research Ethics Committee. The patients/participants provided their written informed consent to participate in this study.

## Author Contributions

MB developed quantification methods, supervised MS support staff, and wrote the manuscript. TW developed quantification methods and wrote the manuscript. AL researched literature and wrote the manuscript. NW undertook volunteer recruitment in Newcastle, coordinated volunteer CRF visits, and supervised CRF support staff. DT and AG undertook volunteer recruitment in Portugal and coordinated volunteer visits and supervised support staff. LL and HP provided QQQ technical support and data generation. JM coordinated project and supervised research in Newcastle University. JD coordinated project, supervised research in Aberystwyth, designed Figures, and wrote the manuscript. All authors contributed to the article and approved the submitted version.

## Conflict of Interest

The authors declare that the research was conducted in the absence of any commercial or financial relationships that could be construed as a potential conflict of interest.

## Supplementary Material

Supporting Data 1 Putative urinary dietary exposure biomarkers from a literature search between the years of 2010–2020.

Supporting Data 2 Foods consumed frequently by the UK population reported to have urine biomarkers and foods used in the MAIN study menu design.

Supporting Data 3 Selection of biomarkers for panel development.

Supporting Data 4 Technical detail of Triple Quadrupole targeted quantification of dietary intake biomarkers including: ionisation mode; chromatography column; retention time selected reaction monitoring (SRM) parameters of collision energy and product ion mass; quantification ion product; limitation of measurement, and concentrations in FMV urine.

Supporting Data 5 Quantile-Quantile (QQ) plots of biomarker concentrations (*n* = 52), where **(A)** First Morning Void (FMV); **(B)** 24 h urine collection.

Supporting Data 6 Urine collection kit characteristics and supplier.

## Abbreviations

AUC: area under the ROC (Receiver Operator Characteristic) curve
BFI: biomarker of food intake
CRN: Clinical Research Network
FFQ: Food Frequency Questionnaire
FMV: First Morning Void
HESI: heated electrospray ionisation
HILIC: Hydrophilic Interaction Liquid Chromatography
HPLC: high-performance liquid chromatography
IAN-AF: Portuguese National Food, Nutrition and Physical Activity Survey
ISRCTN: International Standard Randomised Controlled Trials Number
LC-QQQ-MS: liquid chromatography triple quadrupole mass spectrometry
LoD: limit of detection
logP: partition coefficients
LoQ: limit of quantification
MACCS: Molecular ACCess System
MAIN: Metabolomics at Aberystwyth, Imperial and Newcastle
MDS: multi-dimensional scaling
MRC: Medical Research Council
MRM: multiple reaction monitoring
MS: Mass Spectrometry
NDNS: National Diet and Nutrition Survey
PCA: Principal Components Analysis
QC: Quality Control
RF: Random Forest
RI: refractive index
ROC: Receiver Operator Characteristic
RP: reverse phase
RSD: relative standard deviation
SG: specific gravity
SRM: Selected Reaction Monitoring
UHPLC: Ultra High Performance Liquid Chromatography.
